# Environmental and Temporal Effects on Vocal Activity in a Nocturnal Primate: Implications for Passive Acoustic Monitoring

**DOI:** 10.1002/ajp.70134

**Published:** 2026-03-23

**Authors:** Luke D. Martin, Eva S. Nomenjanahary, Herison Razafimanantsoa, Sylviane Volampeno, Alice M. Richardson, Robert D. Magrath, Alison M. Behie

**Affiliations:** ^1^ School of Archaeology and Anthropology, College of Arts and Social Sciences The Australian National University Canberra Australian Capital Territory Australia; ^2^ Department of Anthropobiology and Sustainable Development University of Antananarivo Antananarivo Madagascar; ^3^ Department of Anthropology University of Colorado Boulder Boulder Colorado USA; ^4^ Mikajy Natiora Association Antananarivo Madagascar; ^5^ Statistical Support Network The Australian National University Canberra Australian Capital Territory Australia; ^6^ Division of Ecology and Evolution, Research School of Biology The Australian National University Canberra Australian Capital Territory Australia

**Keywords:** bioacoustics, *Lepilemur*, primate conservation, remote sensing, surveys, vocal behavior

## Abstract

Passive acoustic monitoring (PAM) is a promising, if underused, technology for primate conservation. Successful PAM requires an understanding of the target species' vocal activity patterns and the factors that influence them, but this information remains scarce for most vocal primates. This is true for sportive lemurs (*Lepilemur* spp.), which are understudied but otherwise excellent candidates for PAM, being highly vocal and threatened. We deployed autonomous audio recorders to measure vocal activity in the Critically Endangered Nosy Be sportive lemur (*Lepilemur tymerlachsoni*), sampling a 4‐h window from twilight each night for two lunar cycles. Our objectives were to identify suitable call types for monitoring, evaluate a user‐friendly automated call detection algorithm, assess temporal variation in vocal activity, and examine how environmental variables and moon illumination influence vocal activity. Automated call detection found an estimated 38% of all target calls but generated a high rate of false positives (96%). Among three call types, “ouah” calls were common and had the highest detection rate (51%), making them suitable target calls. Call rates were highest in the fourth hour following twilight, increased with temperature and moon illumination, and decreased during rainfall. We also observed variation in vocal activity between recording dates and sites, highlighting the need for sufficient temporal and spatial replication. We present recommendations for improving survey design, detection probability, and population inferences from PAM. The recommendations are specific to *L*. *tymerlachsoni* and may guide similar work on other sportive lemurs, although species‐specific differences in vocal behavior and ecology must also be considered.

## Summary

- We measured vocal activity in a nocturnal primate, the Nosy Be sportive lemur (*Lepilemur tymerlachsoni*), for two lunar cycles using autonomous audio recorders.
- Vocal activity increased over the 4‐h sampling window starting at twilight. The lemurs also called more as temperature and moonlight increased, and called less when it rained.
- We present recommendations for improving the design and interpretation of passive acoustic monitoring programs for *L*. *tymerlachsoni*, which may serve as a foundation for similar work with other sportive lemurs.

## Graphical abstract

Passive acoustic monitoring of the Critically Endangered Nosy Be sportive lemur (*Lepilemur tymerlachsoni*) for two lunar cycles revealed that vocal activity (i.e., call rates) increased over a 4‐h sampling window starting at twilight. The lemurs also called more as temperature and moonlight increased, and called less when it rained.

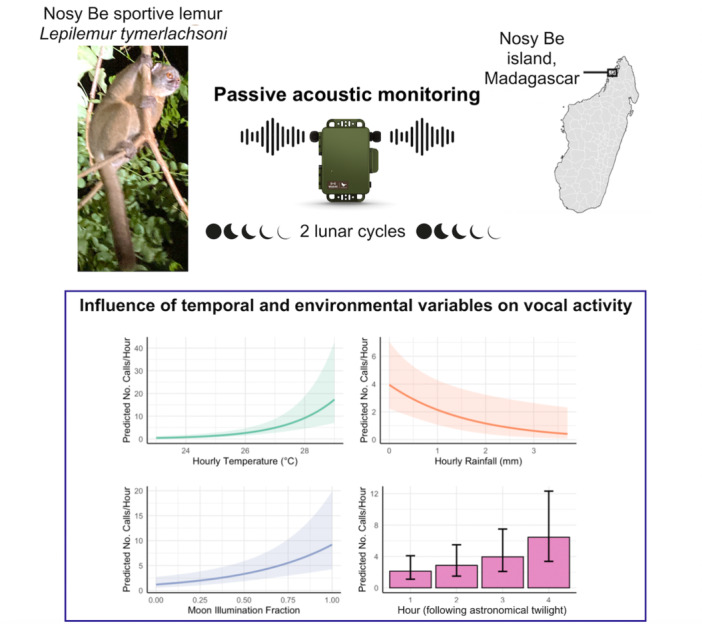

## Famintinana

Ny fanaraha‐maso akostika pasiva (*Passive Acoustic Monitoring na PAM*) dia teknojia mampanantena ho an'ny fiarovana ny primata, na dia mbola tsy dia ampiasaina firy aza. Ny fahombiazan'ny programa PAM rehetra dia miankina indrindra amin'ny fahatakarana ny lamina sy ny haavon'ny asan'ny feo ataon'ireo karazana kendrena ary koa ireo anton‐javatra misy fiantraikany amin'izany. Na izany aza, mbola vitsy ireo fampahalalana ireo ho an'ny ankamaroan'ny primata mampiasa feo. Marina indrindra izany ho an'ny lémurien sportive (*Lepilemur* spp.), izay mbola tsy dia nodinihina firy, nefa kosa tena mety amin'ny PAM satria tena be feo sady tandindomin‐doza mafy. Nametraka fitaovana fandraketana feo mandeha ho azy izahay mba handrefesana ny asan'ny feo ataon'ny lémurien sportive ao Nosy Be (*Lepilemur tymerlachsoni*), karazana sokajiana ho tandindomin‐doza tanteraka. Ny fandraketana dia natao nandritra ny fotoana adiny efatra isaky ny alina, nanomboka tamin'ny takariva, nandritra ny tsingerin'ny volana roa misesy. Ny tanjon'ity fanadihadiana ity dia ny hamantarana ireo karazana antso mety indrindra ho an'ny fanaraha‐maso, ny hanombanana algôritma tsotra sy mora ampiasaina amin'ny fitiliana antso mandeha ho azy, ny handinihana ny fiovaovan'ny asan'ny feo arakaraka ny fotoana, ary ny hijerena ny fiantraikan'ny masontsivana ara‐tontolo iainana sy ny hazavan'ny volana amin'ny asan'ny feo. Ny rafitra fitiliana mandeha ho azy dia nahitana manodidina ny 38% amin'ireo antso kendrena rehetra, saingy niteraka taha avo be amin'ny valiny diso (96%), izay nesorina taorian'ny fanamarinana an‐tanana. Amin'ireo karazana antso telo nodinihina, ny antso “ouah” no hita matetika indrindra ary nahitana taha ambony indrindra amin'ny fitiliana mandeha ho azy (51%), ka nahatonga azy ireo ho antso kendrena mety indrindra ho an'ny fanaraha‐maso. Hitanay fa ambony indrindra ny tahan'ny antso ataon'i *L. tymerlachsoni* amin'ny ora fahefatra aorian'ny takariva. Nitombo izany taha izany rehefa niakatra ny mari‐pana sy ny hazavan'ny volana, ary nihena kosa nandritra ny rotsak'orana. Nisy ihany koa fahasamihafana lehibe teo amin'ny asan'ny feo arakaraka ny daty sy ny toerana nanaovana ny fandraketana, izay manasongadina ny maha‐zava‐dehibe ny famerimberenana ara‐potoana sy ara‐jeografika ampy. Manolotra tolo‐kevitra izahay mba hanatsarana ny famolavolana fanadihadiana, ny vintana hahitana antso, ary ny fanombanana ny mponina amin'ny alalan'ny PAM. Na dia natao manokana ho an'i *L. tymerlachsoni* aza ireo tolo‐kevitra ireo, dia afaka manome fototra ho an'ny fikarohana mitovitovy amin'izany amin'ny lémurien sportive hafa, raha raisina an‐tsaina ihany koa ny fahasamihafan'ny fitondran‐tena ara‐feo sy ny ekôlôjia manokana isaky ny karazana.

**Abbreviations**
PAM: passive acoustic monitoring

## 1 Introduction

Primate vocalizations are rich sources of ecological data, encoding a range of information from and about the sender, including their identity (e.g., species and sex), status or internal state (e.g., fitness and arousal), and environment (e.g., predators and food) (Geissmann and Parsons 2011). From a conservation standpoint, researchers can use conspicuous vocalizations to detect primates and derive key population metrics, including presence/absence, occupancy, distribution, habitat use, and density and abundance (Plumptre et al. 2013; Zwerts et al. 2021). Acoustic surveys can be particularly useful for primates that are otherwise difficult to detect visually, such as nocturnal species. Temporal and environmental variables (e.g., time of day, season, temperature, rainfall, and moon illumination) can affect vocal activity, signal transmission, and detection probability, and should be carefully considered when designing and interpreting acoustic surveys (Bruni et al. 2014; Sugai et al. 2020; Symes et al. 2022). Knowing how these variables affect vocal activity can also be applied in more advanced analyses, like multiple covariate distance sampling and occupancy modeling, to improve detection probability models and make more reliable inferences (Campbell et al. 2019; Marques et al. 2007). Among vocal primates, such influences have mainly been documented in a few diurnal and singing species (e.g., Batist et al. 2022; Clink et al. 2020; Coudrat et al. 2015; Ferrario et al. 2024; Pérez‐Granados and Schuchmann 2021), but for most others, including nocturnal species, the data remain relatively scarce (Gursky and Nekaris 2019).

Passive acoustic monitoring (PAM) involves deploying autonomous audio recorders in the field to monitor wildlife and environments, and is a promising and powerful method for conducting acoustic surveys remotely and non‐invasively (Browning et al. 2017). The ability of these recorders to sample continuously and to be deployed simultaneously across multiple locations also increases the scale and resolution of acoustic data collection. At the same time, it presents a unique opportunity to study spatiotemporal variation in vocal activity and provide new ecological insights (Pérez‐Granados and Schuchmann 2020; Pérez‐Granados et al. 2021). For example, a recent study using 24‐h PAM recordings showed that ostensibly diurnal Guianan red howler monkeys (*Alouatta macconnelli*) vocalize mainly at night (Do Nascimento et al. 2021). However, these strengths come with an attendant “big data” problem: PAM often generates massive acoustic datasets. This makes thorough preparation, optimized data collection, and efficient data processing (e.g., via automated call detection algorithms) highly advantageous (Batist et al. 2024; Dufourq et al. 2021; Heinicke et al. 2015).

Paramount to any successful PAM program is understanding the target species' vocal behavior. This includes knowledge of its vocal repertoire so that detected calls can be attributed accurately to the species, and having information on its vocal activity patterns to guide survey design and interpretation (Sills and Reichmuth 2022; Sugai et al. 2019; Van Parijs et al. 2009). For instance, knowing when a species is most vocal helps researchers maximize detection probability by focusing sampling windows on peak vocal activity periods; this optimizes data collection and promotes cost‐effectiveness, as recording schedules can be shortened, thereby reducing demands on the recorders' battery life and data storage, which in turn reduces the need for field maintenance and allows for longer deployments (Gibb et al. 2019; Sugai et al. 2020). Similarly, knowing that certain environmental conditions, like rain, can suppress vocal activity (e.g., Cheyne 2008; Clink et al. 2020) helps to interpret quiet periods in the data. Ideally then, for successful PAM, key questions must be answered: Which call types are sufficiently common, detectable, and thus suitable to target? When does the target species typically vocalize? And what environmental factors shape its vocal activity?

Despite growing interest, PAM is still underused in primatology (Piel et al. 2022; reviewed in Batist et al. 2024). This seems a missed opportunity. Many primates are both conspicuously vocal and threatened with extinction, making them promising candidates for PAM‐based conservation (Estrada et al. 2017; Markolf et al. 2022). The 25 species of sportive lemurs (genus *Lepilemur*), all endemic to the Indian Ocean island of Madagascar, are an excellent example, being highly vocal and highly threatened (Méndez‐Cárdenas et al. 2008; Mittermeier et al. 2008, 2023). As strictly nocturnal primates often found in remote areas (Wilmet et al. 2014), they can also be challenging to observe and survey using traditional visual methods. This is true of the Critically Endangered Nosy Be sportive lemur (*Lepilemur tymerlachsoni*), much of whose limited range is difficult to access and traverse, particularly at night (Martin et al. 2025a). Perhaps as a consequence, sportive lemurs remain understudied (Ramananjato et al. 2025), and there is insufficient information on their vocal activity to usefully guide PAM programs.

As a first step to addressing this, we used autonomous recorders to monitor vocal activity in *Lepilemur tymerlachsoni*, measured as the number of calls detected by an automated call detection algorithm and confirmed through manual verification. We recorded a 4‐h window from twilight each night for two lunar cycles during the wet season, to sample the time of year and time of night that sportive lemurs are generally most active, including vocally (Henry 2023; Mahaboubi et al. 2015; Mandl et al. 2019; Nash 1998; Russell 1977; Warren and Crompton 1997). Our objectives were to: (1) identify the most suitable call types for monitoring, based on their commonness and detectability, (2) evaluate the performance of a readily available and user‐friendly automated call detection algorithm, (3) assess temporal variation in vocal activity within the 4‐h sampling window, and (4) examine the influence of temperature, rainfall, and moon illumination on vocal activity.

In primates, activity levels, including vocal activity, reflect trade‐offs that are influenced by energy expenditure, environmental conditions, and predation risk, among other factors (Erkert et al. 2012; Rahalinarivo et al. 2023; Rode‐Margono and Nekaris 2014). For sportive lemurs, predation presents a significant risk. Known predators of *Lepilemur tymerlachsoni* include several raptors and domestic dogs (Andrews et al. 1998), while owls, boas, and introduced forest cats are also likely threats (Dröscher and Kappeler 2014; Scheumann et al. 2007). Notably, however, native carnivores like the fosa (*Cryptoprocta ferox*), which normally prey upon lemurs, are absent from Nosy Be. Increased moonlight typically suppresses activity in nocturnal mammals due to such predation risk (so‐called lunar phobia)—a phenomenon widely attributed to predators' increased ability to detect prey in brighter conditions (Prugh and Golden 2014). Yet many nocturnal primates, including some sportive lemurs (Nash 2007; but see Campera et al. 2019), exhibit the opposite trend (so‐called lunar philia), tending to be more active, including vocally, on brighter nights, possibly because of the relative importance of vision in primates for foraging and predator detection (Prugh and Golden 2014). Vocalizing can also be energetically costly (Ryan 1988), and is likely to be especially so for sportive lemurs, considering their very low resting metabolic rates and low‐quality, folivorous diets (Nash 1998; Schmid and Ganzhorn 1996). Colder temperatures and rainfall can increase thermoregulatory costs, which may reduce the energy available for vocalizing and prompt energy‐conserving behavioral modifications, and rainfall can cause acoustic masking that affects signal propagation, potentially decreasing vocal activity (Cowlishaw 1996; Dinsmore et al. 2021; Lengagne and Slater 2002; Mandl et al. 2018). The suppressive effects of these factors may be immediate or delayed; for example, the early morning songs of some gibbons are less frequent and start later when it is raining (Cheyne 2008) or when there has been rain in the previous night (Clink et al. 2020). Based on this rationale, we predict that: (1) vocal activity will be higher with increased moon illumination, consistent with general patterns in nocturnal primates, and (2) vocal activity will be lower with colder temperatures and during rainy conditions, which we hypothesize to result from behavioral modifications associated with increased thermoregulatory costs and potentially reduced signal propagation.

By identifying common call types, their detectability, optimal sampling windows, and environmental influences, we aim to guide future acoustic surveys—and particularly PAM programs—for *Lepilemur tymerlachsoni* and provide a template for similar work on other sportive lemurs, ultimately contributing to more accurate assessments of key population metrics for conservation management.

## 2 Methods

### 2.1 Ethics Statement

This research adhered to protocols approved by the Australian National University's Animal Experimentation Ethics Committee (#A2022/30) and the national laws of Madagascar and Australia. The Ministère de l'Environnement et du Développement Durable of Madagascar granted research permission (#346/22/MEDD/SG/DGGE/DAPRNE/SCBE.Re). We followed the American Society of Primatologists (ASP) Principles for the Ethical Treatment of Non‐Human Primates and its Code of Best Practices for Field Primatology.

### 2.2 Study Population

The study took place in the Lokobe National Park region of the island of Nosy Be in northwestern Madagascar. This area features protected primary humid evergreen forest (characterized by tall, large trees and minimal invasive vegetation), unprotected secondary forest (regrowth areas with smaller, denser trees and invasive vegetation), and small‐scale agricultural plantations (e.g., ylang‐ylang, vanilla, and rice) interspersed with secondary forest fragments (Tinsman et al. 2022). The climate is tropical, with a hot, wet season (November–April) and a cool, dry season (May–October) (Macron et al. 2016). Local temperature and rainfall data were collected during the study period (see Environmental Variables below).

**FIGURE 1:**
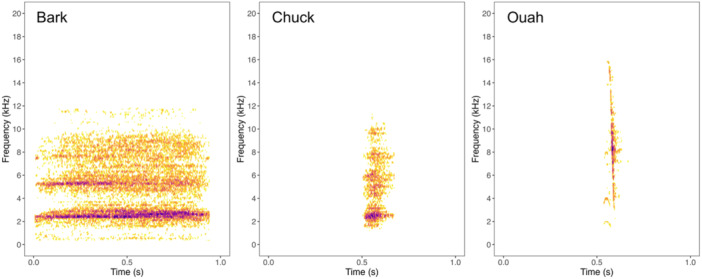
Representative spectrograms of the bark, chuck, and ouah calls of *Lepilemur tymerlachsoni*. Spectrograms were created using the *seewave* R package (Sueur et al. 2023) (Hann window = 512 samples, discrete Fourier transform size = 512, hop size = 153.6 samples, grid spacing = 86.1 Hz, overlap = 70%).

The Nosy Be or Hawks' sportive lemur (*Lepilemur tymerlachsoni*) is a microendemic of Nosy Be, and is distributed across the various habitats of the Lokobe region (Louis et al. 2020; Sawyer et al. 2015; Tinsman et al. 2022). We focused on three common call types: the “bark,” “chuck,” and “ouah.” These call types represent > 85% of vocalizations recorded from *L*. *tymerlachsoni* in a concurrent study (Martin et al. 2026), and are shared with other *Lepilemur* species (e.g., Mandl et al. 2019; Rasoloharijaona et al. 2006). Barks are harsh, loud, relatively long, low‐frequency calls; chucks are short, harsh calls of moderate bandwidth and low peak frequency; ouahs are short, broadband calls with a characteristic harmonic onset and downward frequency sweep (Figure 1). We based our analyses on individual calls, defined here as single acoustic units separated by a silent gap. Each call type is usually produced singly, but also sometimes produced as rapid sequences of two or more calls, or as extended bouts (e.g., > 10 min) (Mandl et al. 2019; Martin et al. 2026).

### 2.3 Acoustic Recordings

We established 10 acoustic recording sites in the Lokobe region. The sites were evenly distributed using a systematic grid with a random start point, encompassing much of the species' restricted extent of occurrence (72 km^2^) and remaining forest habitat (37.3 km^2^) (Louis et al. 2020; Martin et al. 2025a, 2025b). At each site, we deployed an autonomous audio recorder (Wildlife Acoustics Inc., Song Meter SM4) 2 m above ground and mounted to a tree in a forested area, taking care to avoid potential sources of acoustic interference (e.g., rivers and villages). Recordings were made during the wet season from February 1 to April 5, 2023. We scheduled recordings for 4 h each night, starting at astronomical twilight (c. 7–7:30 p.m.), to sample expected periods of high vocal activity and to balance data collection with the practical and logistical limitations of recorder battery life, data storage, and field maintenance. Recorders were deployed for 24–63 days (mean: 54), spanning two lunar cycles. The Song Meter SM4 recorders used two built‐in omnidirectional microphones and recorded stereo uncompressed (.wav) 1‐h sound files (44.1 kHz, 16‐bit sampling, 16 dB gain, 26 dB preamplifier gain, 220 Hz high‐pass filter).

**FIGURE 2:**
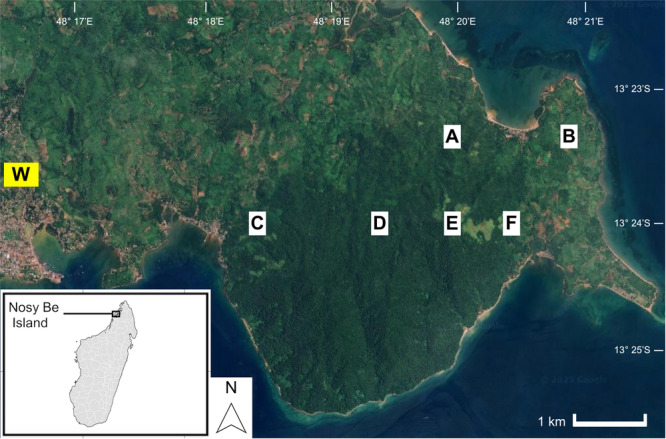
Study area in the Lokobe region of the island of Nosy Be, northwestern Madagascar, showing the locations of the six acoustic recording sites (A–F) and weather station (W).

We analyzed recordings from six recorders, each separated by > 850 m (Figure 2). Recordings from the other four sites were excluded due to insufficient data: at three sites no calls were detected, and at one site only 32 total calls were detected over 62 nights, providing no or limited information for modeling vocal activity patterns at those sites. Some recorders collected an additional 1 h of data before astronomical twilight, however we only included the 4‐h period beginning at astronomical twilight in our analysis; this extra hour was excluded to ensure consistency across recorders and because it was often saturated with loud insect noise across a large frequency range, which compromised automated call detection.

### 2.4 Environmental Variables

Total hourly rainfall (mm) and mean hourly air temperature (°C) data were collected from a weather station (Digitech, IC‐XC0430) located approximately 3 km from the study area (Figure 2). The weather station's off‐site location was necessary for daily inspection of the data, as accessing the recording sites was difficult and intermittent. These hourly measurements were synchronized with the 5‐h period (beginning 1 h prior to astronomical twilight) used for the passive acoustic recordings. The daily fraction of moon illumination was obtained from the Astronomical Applications Department of the U.S. Naval Observatory (https://aa.usno.navy.mil/data/MoonFraction.html). Wind may also affect vocal activity in primates (Williams 2017), but as wind speeds were consistently negligible ( < 1 km/h) throughout the survey period (see Martin et al. 2025a) we did not include this variable in our analysis.

### 2.5 Automated Call Detection and Manual Verification

Spectrograms of the left microphone (channel 1) acoustic recordings were scanned for bark, chuck, and ouah calls using the band‐limited energy detector available in Raven Pro 1.6.4 (K. Lisa Yang Center for Conservation Bioacoustics, 2023). This detector works by identifying signal sections that exceed a user‐specified signal‐to‐noise ratio threshold within defined time and frequency bands. It requires input parameters for minimum and maximum frequency (Hz), minimum and maximum duration (s), and minimum separation (s)—which defines the minimum gap allowed between candidate calls (Charif et al. 2010; Mills 2000).

We ran a separate detector for each of the three call types, using conservative input parameters to prioritize detecting target calls at the expense of a higher number of false positives (i.e., prioritizing recall over precision; Table 1). Initial frequency and duration parameters were based on the fifth and 95th percentiles of acoustic measurements from a concurrent *Lepilemur tymerlachsoni* vocal repertoire study (Martin et al. 2026). These were rounded to the nearest 100 Hz and 0.01 s, respectively. After preliminary analysis, we doubled the maximum duration parameter, which improved call detection. Minimum separation was uniformly set to 0.01 s. We applied a signal‐to‐noise ratio (SNR) threshold of 10 dB (i.e., how far above the background noise a sample must be to be considered a signal) and a minimum occupancy of 50% (i.e., the percentage of samples within the detector's range that must exceed this SNR threshold) (Charif et al. 2010). Following Charif et al. (2010), for noise power estimation we set the block size to 3 times the maximum duration and the hop size to double the maximum duration, using the default 20th percentile noise parameter. We noted the automatically recorded time taken for each detector to scan each 1‐h recording.

**TABLE 1:** Detector input parameters for each call type.

Call type	Minimum frequency (Hz)	Maximum frequency (Hz)	Minimum duration (s)	Maximum duration (s)	Minimum separation (s)
Bark	600	11,000	0.3	2.7	0.01
Chuck	800	14,000	0.07	0.48	0.01
Ouah	600	19,700	0.08	0.32	0.01

An experienced analyst (LDM) manually reviewed all candidate calls identified by the automated detectors. Each candidate call was visually inspected on the spectrogram and listened to as needed in Raven Pro. This review distinguished true positives (correctly identified target calls) from false positives (incorrectly identified other sounds) based on spectrogram images and sound clips of call type exemplars and graded variants compiled in a concurrent vocal repertoire study (Martin et al. 2026). The final count of true positives was then used to model the effects of temporal and environmental variables on vocal activity and to partly evaluate detector performance. We recorded the time taken to manually review the candidate calls identified by each detector for each 1‐h recording.

We used precision and recall as our metrics to evaluate detector performance. Precision is the proportion of detections that are correct; recall is the proportion of target calls that are detected (Knight et al. 2017). Precision was calculated using the true and false positive counts from the manual review and the formula: precision = true positives/(true positives + false positives). To estimate recall, LDM manually inspected 10 randomly selected 1‐h sound files per recorder to determine true positives and false negatives (target calls missed by the detectors) within the sample. Recall was then estimated for the sample using the formula: recall = true positives/(true positives + false negatives).

### 2.6 Statistical Analysis

Generalized linear mixed models (GLMMs) were fitted in R version 4.4.0 (R Core Team 2024) using the *glmmTMB* package (Brooks et al. 2017) to model the effects of temporal and environmental variables on vocal activity. We excluded all 1‐h acoustic recordings with > 4 mm rainfall (c. 7% of all data) because heavy rain obscures most sounds. We also excluded observations with missing data for any other relevant variables (e.g., due to weather station malfunction), which resulted in 996 hourly observations for model fitting. We note here that calls were treated as independent events in our GLMMs, despite vocal activity sometimes occurring in non‐independent clustered bouts (see further discussion in Limitations below).

### 2.7 Model Specification and Selection (Primary Analysis)

The response variable was the total number of detected calls (bark, chuck, and ouah combined) per 1‐h recording. For the model structure, we considered candidate GLMM families comprising Poisson and negative‐binomial (linear and quadratic parameterization) distributions with log link functions, both with and without zero‐inflation. These distributions are appropriate for ecological count data, which often exhibit overdispersion and may also be zero‐inflated (Brooks et al. 2017; Martin et al. 2005). For fixed effects, we included hourly temperature (°C), hourly rainfall (mm), daily moon illumination fraction, and day number (the *i*th day from the first recording day, to test for intra‐seasonal trends) as continuous predictors. All of these continuous predictors were Z‐score standardized (mean = 0 and SD = 1) prior to analysis. Additionally, hour was included as a categorical predictor variable (four levels representing consecutive 1‐h periods starting at astronomical twilight). To assess multicollinearity among predictors, we calculated variance inflation factors (VIFs) using the package *performance* (Lüdecke et al. 2021). As all VIFs were low, we retained all predictors. We included date and recorder ID as random effects to account for variability between recording dates and sites.

We first identified the optimal response distribution and zero‐inflation structure from a set of candidate global models (representing each distribution and zero‐inflation combination and containing all fixed and random effects) based on the lowest Akaike Information Criterion (AIC) value (Akaike 1973). We assessed this model's goodness‐of‐fit by inspecting QQ‐plots and performing nonparametric dispersion, zero‐inflation, outlier (bootstrapped method), and categorical predictor tests of the simulated residuals using the *DHARMa* package (Hartig 2022). Following this, we fitted models with the random effects and all possible combinations of the fixed effects, using the optimal distribution and zero‐inflation structure. We ranked these models by AIC and selected the model with the lowest AIC (ΔAIC = 0) as our final model. For significant categorical fixed effects, we performed post hoc pairwise comparisons using Tukey's tests with the *emmeans* package (Lenth 2024). To visualize predicted population‐level effects for each fixed effect included in the final model, we calculated estimated marginal means using *emmeans*. These predictions were back‐transformed to the original count scale (number of calls per hour), with other fixed effects held at their mean or reference levels, and are presented in the results with 95% confidence intervals.

### 2.8 Sensitivity Analysis

To assess potential non‐linear effects of the continuous predictors, we fitted a generalized additive mixed model (GAMM) using the *mgcv* package (Wood 2025). The GAMM used the same distribution family and the same set of predictors as the final GLMM in the primary analysis. Smooth terms were included for the continuous predictors (temperature, rainfall, and moon illumination), and hour was included as a categorical predictor. Random‐effect smooths were included for date and recorder ID. We checked model diagnostics and inspected plots of the modeled smooth functions using *mgcv*, and compared effect directions and statistical significance with those from the primary GLMM.

### 2.9 Preceding Rainfall (Secondary Analysis)

We conducted a separate secondary (GLMM) analysis to investigate the delayed or lagged effects of rainfall on vocal activity, independent of current conditions. This analysis was performed on a subset of the data for which a binary categorical predictor, preceding rainfall (indicating the presence [> 0 mm] or absence of rain in the immediately preceding 1‐h recording period), was available. Separating the analyses was necessary because directly including this variable in the primary analysis would have substantially reduced the number of observations, as data for preceding rainfall were not consistently collected for all first hourly recording blocks. We compared two models: a base model that included the same fixed and random effects as the final model in the primary analysis (including hourly rainfall), and a second model that also included preceding rainfall. Both models used the same distribution family (and any zero‐inflation) selected in the primary analysis. We calculated VIFs and evaluated the relative fit of the models using AIC, and assessed the significance of preceding rainfall's effect using a likelihood‐ratio test.

All statistical tests were performed at an alpha level of 0.05.

## 3 Results

### 3.1 Automated Call Detection

The automated detectors processed 1120 total hours of acoustic recordings, independently scanning each 1‐h recording for bark, chuck, and ouah calls. The entire automated scanning process (3360 cumulative hours: 1120 h × 3 scans, one for each call type) was completed in 5 h and 24 min, averaging about 6 s of processing time for each call type per 1‐h recording.

The automated detectors performed with modest recall but very low precision, identifying a large volume of candidate calls that required manual verification (Table 2). The detectors identified a total of 316,280 candidate calls across call types. Of these, 12,988 (4%) were manually verified as true positive detections, and the remainder (96%) as false positives, meaning the precision across call types was 0.04. The detectors identified 38% of all target calls within the random subsample of 60 manually‐screened 1‐h acoustic recordings (10 from each of the six recorders), with variation among call types (Table 2). Estimated recall was thus 0.38. Among all true positive detections, chuck calls were the most frequent (45%), followed by ouah calls (40%) and bark calls (15%). The detectors identified a mean of 11.58 true positives per 1‐h recording, which is the sum of the mean true positives for each call type. Manual verification of all candidate calls required a total of 27 h and 57 min.

**TABLE 2:** Summary of automated detector performance and manual verification effort.

Call type	Candidate calls	True positives	Precision	Recall	% of total true positives	Mean candidate calls/1‐h recording	Mean true positives/1‐h recording	Mean manual review time (s)/1‐h recording
Bark	54,671	1935	0.04	0.43	15	48.81	1.73	19
Chuck	161,363	5874	0.04	0.22	45	144.07	5.23	44
Ouah	100,246	5179	0.05	0.51	40	89.51	4.62	26
All	316,280	12,988	0.04	0.38	100	287.01	11.58	—

*Note:* Total acoustic recordings processed: 1120 h. Total automated scanning time across call types: 3360 h (1120 h × 3 scans, one for each call type) completed in 5 h and 24 min. Total manual verification effort: 27 h and 57 min. Precision is the proportion of detections that are correct, and recall is the proportion of target calls that are detected.

### 3.2 Factors Influencing Vocal Activity

We fitted GLMMs to our vocal activity data and based model selection on AIC values and goodness‐of‐fit tests. The selected GLMM was the negative binomial (quadratic parameterization) without zero‐inflation. Diagnostic plots of simulated residuals showed a good fit, with no significant deviations from uniformity (*p* = 0.46), no overdispersion (*p* = 0.57), no zero‐inflation (*p* = 0.94), and no significant outliers (*p* = 0.16). The categorical predictor test for hour also showed no issues (all individual category *p*‐values > 0.32). Collinearity among the predictors was low (VIFs ≤ 1.54). The final model included temperature, rainfall, moon illumination, and hour as significant predictors of vocal activity, while day number was not included.

**FIGURE 3:**
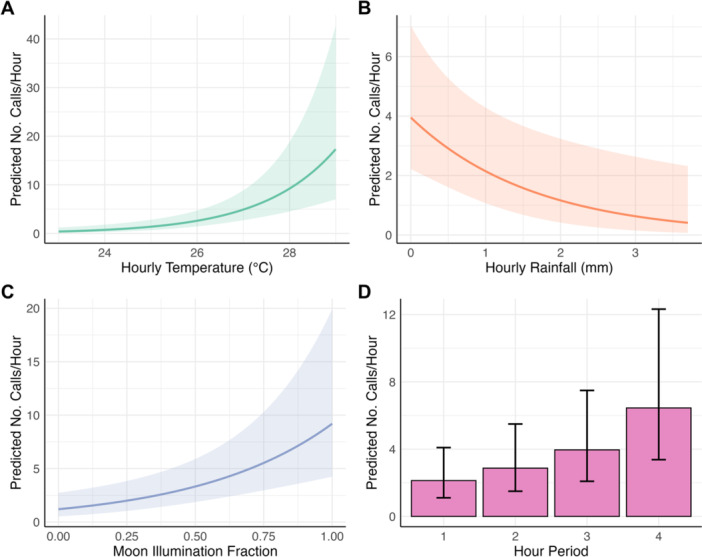
Predicted effects of temporal and environmental covariates on *Lepilemur tymerlachsoni* call rates per hour from a GLMM. The *y*‐axes represent the predicted number of calls per hour (bark, chuck, and ouah combined), with other covariates held at their mean or reference levels. (A) Hourly temperature (°C). (B) Hourly rainfall (mm). (C) Moon illumination fraction. (D) Hour period (four 1‐h periods commencing at astronomical twilight). Shaded areas and error bars represent 95% confidence intervals.

*Lepilemur tymerlachsoni* call rates increased with temperature and moon illumination, and decreased with rainfall (Table 3, Figure 3). Specifically, a one standard deviation increase in hourly temperature (equivalent to 1.26°C) was associated with an increase in the expected number of calls by a factor of 2.21 (*p* < 0.001). Conversely, a one standard deviation increase in hourly rainfall (equivalent to 0.59 mm) was associated with a decrease in expected calls by a factor of 0.69 (*p* < 0.01). Moon illumination also had a significant positive effect, with a one standard deviation increase (equivalent to 0.35 fraction) leading to a 2.05‐fold increase in expected calls (*p* < 0.001).

**TABLE 3:** Generalized linear mixed model results for factors influencing *Lepilemur tymerlachsoni* vocal activity.

Predictor	Estimate (log scale)	SE	Z‐value	*p* value	Fold change (exp(Estimate))
**Fixed effects** (Intercept)					
	0.734	0.334	2.199	< 0.05	2.08
Temperature (SD)	0.792	0.178	4.448	< 0.001	2.21
Rainfall (SD)	−0.374	0.144	−2.601	< 0.01	0.69
Moon illumination (SD)	0.718	0.2	3.594	< 0.001	2.05
Hour (relative to hour 1)					
‐ Hour 2	0.299	0.242	1.236	0.216	1.35
‐ Hour 3	0.62	0.255	2.428	< 0.05	1.86
‐ Hour 4	1.109	0.272	4.083	< 0.001	3.03
**Random effects**	**SD**				
Date (Intercept)	1.241				
Recorder ID (Intercept)	0.53				

*Note:* The selected GLMM employed a negative binomial (quadratic parameterization) distribution with a log link. Tabulated *p*‐values for the hour categorical predictor are from the final model output, but further pairwise comparisons used Tukey's adjustment (see text for full comparisons).

Vocal activity increased by the hour (Table 3, Figure 3). The model coefficients (relative to hour 1, i.e., the first hour after astronomical twilight) indicated that vocal activity significantly increased during hour 3 (1.86‐fold increase, *p* < 0.05) and hour 4 (3.03‐fold increase, *p* < 0.001). The difference for hour 2 (1.35‐fold increase, *p* = 0.216) was not statistically significant. However, post hoc pairwise comparisons using Tukey's adjustment showed that only call rates in hour 4 were significantly higher than in both hour 1 (*p* < 0.001) and hour 2 (*p* < 0.01). Calls in hour 3, despite their initial significance relative to hour 1, were not significantly different from hour 1 at the adjusted level (*p* = 0.072). Other pairwise comparisons (hour 1 vs. hour 2, hour 2 vs. hour 3, and hour 3 vs. hour 4) also showed no significant differences (*p* > 0.14).

There was substantial daily variation in vocal activity and measurable, though less pronounced, differences between recording sites. Specifically, this variability was observed between recording dates (SD = 1.241) and, to a lesser extent, among individual recorders (SD = 0.53).

### 3.3 Sensitivity Analysis

The GAMM results were consistent with those of the GLMM. Temperature (effective degrees of freedom [edf] = 2.30, *χ* ^2^ = 28.27, *p* < 0.001) and moon illumination (edf = 1.00, *χ* ^2^ = 13.50, *p* < 0.001) had significant positive effects on call rates, while rainfall (edf = 2.26, *χ* ^2^ = 12.90, *p* < 0.01) had a significant negative effect. Plots of the smooth terms indicated only modest non‐linearity for temperature and rainfall, with monotonic relationships across the observed range that were similar to GLMM predictions, whereas the effect of moon illumination was effectively linear. Call rates, relative to hour 1, increased significantly during hour 3 (estimate = 0.574, *p* < 0.05) and hour 4 (estimate = 1.076, *p* < 0.001), but not during hour 2 (estimate = 0.303, *p* = 0.169), consistent with the GLMM. Diagnostic checks revealed no issues, and the model explained 39.1% of the deviance. Accordingly, the sensitivity analysis did not alter the conclusions drawn from the GLMM about the effects of the predictors.

### 3.4 Effects of Preceding Rainfall

Vocal activity was not significantly affected by preceding rainfall. For this secondary GLMM analysis, we used a reduced dataset of 901 hourly observations, adding a binary predictor for the presence of rainfall in the immediately preceding 1‐h recording block to the final model. Multicollinearity among fixed effects was low (VIFs ≤ 1.68). A likelihood‐ratio test comparing the two models (one with the preceding rainfall predictor on the reduced dataset and one without) revealed that the inclusion of preceding rainfall did not significantly improve the model's fit (*χ* ^2^ = 2.42, *p* = 0.12). The difference in AIC was negligible (model with preceding rainfall AIC = 4178.89; base model AIC = 4179.31). The estimated effect of preceding rainfall was a multiplicative factor of 0.57 (*p* = 0.115), suggesting a reduction in vocal activity when it rained recently, though this effect was not statistically significant.

## 4 Discussion

Time of night, weather, and moonlight affect vocal activity in *Lepilemur tymerlachsoni*. Specifically, we found that *L. tymerlachsoni* vocal activity increased over the 4‐h period following astronomical twilight, being highest in the fourth hour. Vocal activity also increased with temperature and moon illumination and decreased with rainfall. These findings improve our understanding of sportive lemur vocal behavior and ecology. Automated call detection using Raven Pro's band‐limited energy detector, in conjunction with rapid manual verification of candidate calls, efficiently processed our large acoustic dataset, and despite a low proportion of correct detections (i.e., low precision), the detectors still showed a reasonable detection rate (i.e., moderate recall) that would likely be sufficient for many research objectives. Below, we provide recommendations for improving survey design, detection probability, and population inferences from PAM, which are specific to *L*. *tymerlachsoni* but may also serve as a template for similar work on other sportive lemurs.

Automated call detection with manual verification efficiently processed our acoustic recordings. Of the three targeted call types, chucks and ouahs were the most frequently detected calls. Ouahs had the highest detection rate (recall: 0.51), possibly due to their acoustically distinct, higher frequency range. While the proportion of correct detections was very low (precision: 0.04), there is commonly a trade‐off between precision and recall when using automated detection algorithms (Knight et al. 2017; Teixeira et al. 2024). Low precision is not necessarily a problem, depending on resources and the research objectives, and provided it is combined with efficient manual verification and satisfactory recall. In our case, candidate calls were rapidly verified in Raven Pro (Table 2), which helped offset the large numbers of false positives. In total, automated scanning and manual review of a cumulative 3360 h of acoustic data (1120 hourly recordings × 3 scans, one for each call type) took approximately 33 h—just a fraction (c. 3%) of the total length of the recordings.

The Raven Pro detector's performance, when combined with this rapid manual verification workflow, makes it suitable for many research purposes, especially when the primary goal is to confirm presence (e.g., species richness and occupancy modeling). In such cases, verification can usually stop once a true positive is confirmed, which further speeds up the process. For other purposes, though, such as density estimation via acoustic distance sampling (e.g., cue counting), the manual verification needed for an accurate count may be too onerous given such low precision, and the modest recall may be inadequate, as such methods assume calls are detected with certainty at zero distance from the transect line or point or, for PAM surveys, at the recorder itself (Buckland et al. 2001; Martin et al. 2025a). Of course, the performance of the detector depends on the particular soundscape (almost all of the many false positives in our case were attributable to the nightly insect chorus), as well as the choice of input parameters. Precision could potentially be improved, for example, by focusing the detector's minimum and maximum frequency parameters on the parts of the calls with the most energy and which degrade the least (rather than using the conservative fifth and 95th percentiles, as we did), although this may reduce recall. While Raven Pro remains a popular and user‐friendly choice, with a low learning curve for detector setup and operation (Duan et al. 2013; Priyadarshani et al. 2018; Zambolli et al. 2022), more sophisticated machine learning tools are becoming increasingly available and accessible, and are likely to perform better at call detection (Cauzinille et al. 2024; Dufourq et al. 2021; Ravaglia et al. 2023). Overall, the ouah call appears to strike the best balance of being common, acoustically distinct, and detectable (highest recall), making it the most suitable target call for future PAM programs. Chucks could also be a suitable target call, given they too are very common. In our experience, the ouah and chuck detectors often detected the other call type, probably due to some overlap in their acoustic properties (Table 1), which suggests that targeting both in tandem could increase overall detections. The low recall for chucks (0.22) may reflect a limitation of the band‐limited energy detector rather than any inherent detectability of the call type itself, and should therefore not be seen as a barrier to targeting chucks with a more advanced detector.

Our findings on the temporal patterns of *Lepilemur tymerlachsoni* vocal activity establish a sound basis for optimizing PAM recording schedules. Vocal activity increased throughout the 4‐h period following astronomical twilight, being highest in the fourth hour (Figure 3). This is similar to observations in Milne‐Edwards' sportive lemur (*L*. *edwardsi*), where nightly vocal activity peaks in the hour before midnight (Henry 2023; see also Warren and Crompton 1997). Targeting this period offers real practical value for monitoring programs. First, it maximizes detection probability. Second, it can improve efficiency by allowing for shortened or duty‐cycled recording schedules, rather than having to record for the entire night. This strategy reduces demands on a recorder's battery life, data storage, and the need for field maintenance, as well as subsequent data processing. That being said, while this targeted approach optimizes detection on a given night, comprehensive monitoring efforts should still factor in the need for sufficient replication, as we discuss further below. A related avenue for future research is to explore the impacts of anthropogenic noise on *Lepilemur* vocal communication, especially during peak activity periods. Little is known about this topic for sportive lemurs, yet there may be broad conservation implications given the importance of vocal signaling in some species and the proximity of human settlements to their habitats (Duarte et al. 2018; Madliger 2012; Rasoloharijaona et al. 2010; Santos et al. 2017; Teixeira et al. 2019).

The positive relationship between vocal activity and moon illumination has implications for both understanding sportive lemur ecology and designing effective PAM programs. This lunar philic behavior is consistent with our predictions and general activity patterns observed in other nocturnal primates (e.g., Azara's night monkey, *Aotus azarae*, Fernandez‐Duque 2003; Gursky's spectral tarsier, *Tarsius spectrumgurskyae*, Gursky 2003; Tanzania coast dwarf galago, *Paragalago zanzibaricus*, Nash 1986; Southern pygmy loris, *Xanthonycticebus pygmaeus*, Starr et al. 2012), and contrasts with the lunar phobia typical of nocturnal non‐primate mammals (Prugh and Golden 2014). Moonlight's influence on activity in sportive lemurs as a group is, however, mixed. While there is some evidence linking increased moon illumination to vocal activity in the white‐footed sportive lemur (*Lepilemur leucopus*) (Nash 2007), Madame Fleurette's sportive lemur (*L. fleuretae*) reduces its overall activity levels during periods of high luminosity (Campera et al. 2019). The variation among species in the effect of lunar illumination might be due, in part, to variation in predation risk. For instance, the study site for *L. fleuretae* harbors a “full set of predators” (Campera et al. 2019), including the cathemeral fosa, which may contribute to that species' lunar phobia, whereas the fosa and other native carnivores are absent from Nosy Be. This could mean *L*. *tymerlachsoni* perceives relatively lower predation risk on brighter nights, which may contribute to its lunar philic vocal behavior. However, predation risk is unlikely to be the sole explanation, as other lunar philic primates, and even *L*. *leucopus*, are still exposed to high predation pressure (Nash 2007). A further possible explanation relates to population density. There is evidence that *Lepilemur* species living in high densities are more vocal than those in low densities, as vocal signaling is thought to be favored with the increased competition for resources and where there is a higher probability of calls being received by conspecifics (Rasoloharijaona et al. 2010). On brighter nights in high‐density populations, it is possible that improved visibility facilitates visual contact with conspecifics that could initiate calling (Nash 2007). Consistent with this, densities of *L*. *leucopus* and *L*. *tymerlachsoni* are around 2–4 times higher than *L*. *fleuretae* (Campera et al. 2023; Martin et al. 2025a; Ralison 2006). Further behavioral studies are needed to better understand these underlying mechanisms. Regardless, these results suggest that moon illumination should be considered when designing and interpreting PAM surveys of sportive lemurs. Specifically for *L*. *tymerlachsoni*, brighter moon phases lead to increased vocal activity and thus higher detection probabilities.

*Lepilemur tymerlachsoni* vocal activity increased with higher temperatures, consistent with our prediction. Higher temperatures can reduce the energetic demands of thermoregulation, thereby freeing up more energy to be directed toward energetically costly behaviors like vocalizing (Cowlishaw 1996; Erb et al. 2016; Ryan 1988). Such an energy allocation strategy fits within the well‐documented behavioral and physiological adaptations of lemurs to manage energy in their harsh, unpredictable environments (Wright 1999), and is particularly relevant for sportive lemurs, given their extremely low resting metabolic rates and low‐quality, folivorous diets (Ganzhorn 1993; Nash 1998; Schmid and Ganzhorn 1996; Warren and Crompton 1997). Higher ambient temperatures might also theoretically improve call transmission, though the relationship is complex (Waser and Waser 1977), and if so, potentially affect detection probability (Yip et al. 2025). Energetic benefits, however, seem the most likely explanation. A similar positive relationship between temperature and vocal activity has been documented in other lemurs (indri, *Indri indri*, Ferrario et al. 2024) and primates (e.g., diana monkey, *Cercopithecus diana*, Kalan et al. 2015; chimpanzee, *Pan troglodytes*, Piel 2018; Kloss' gibbon, *Hylobates klossii*, Whitten 1982). Our results are further supported by broader seasonal patterns, as activity levels, including vocal activity, in other sportive lemur species dramatically decrease during Madagascar's cool, dry season, although this is perhaps also related to the breeding cycle (Dröscher and Kappeler 2014; Mandl et al. 2018, 2019).

As predicted, *Lepilemur tymerlachsoni* vocal activity decreased with rainfall. This is consistent with observations in other primates, including white‐cheeked gibbons (*Nomascus* sp., Coudrat et al. 2015), black‐and‐white ruffed lemurs (*Varecia variegata*, Batist et al. 2022), and indris (Ferrario et al. 2024). This decrease may reflect calling suppression from two main factors: thermoregulatory costs and acoustic masking. First, rain can prompt sportive lemurs to seek shelter, engage in social huddling, and reduce travel distances to mitigate increased thermoregulatory costs resulting from rain‐affected fur insulation (Mandl et al. 2018), and this redirection of their behavior may curtail vocal activity. Second, rain might disincentivize calling due to the masking effect of increased background noise (Dooley et al. 2012; Schneider et al. 2008). At the same time, acoustic masking may also compromise automated call detection and thus have contributed to the observed decrease (Symes et al. 2022; Whisson et al. 2021), notwithstanding that we excluded recordings above a heavy rain threshold. Interestingly, our secondary analysis found that preceding rainfall (the presence of rain in the immediately preceding 1‐h recording block) did not significantly affect vocal activity, though it did show a negative trend. This suggests that any suppressive effect of rainfall on vocal activity is largely immediate, with any lasting or lagged impact proving too subtle or brief to be detected in subsequent hourly measurements. Future studies using 24‐h rainfall data could test whether related measures (e.g., total daily rainfall and time since rain) influence vocal activity. In any case, to properly account for rainfall in PAM programs, it is important that rainfall data be collected alongside acoustic recordings so that its effects can be modeled and heavy rain periods managed.

The observed variability in vocal activity between recording dates and recording sites highlights the need for sufficient temporal and spatial replication in PAM designs (Sugai et al. 2020). Where replication is insufficient, it can lead to inaccurate results and conclusions. For instance, we occasionally observed “silent” recording nights, even at sites where lemurs were known to be present, which implies they do not always vocalize. Short‐term surveys could wrongly interpret such quiet periods as species absence, when there might simply be a temporary lull in activity. Deploying too few recorders could lead to the same inaccurate conclusions at a landscape level, as the species might be present at un‐sampled locations. On the other hand, limited sampling could generate positive bias in some population metrics if it happens to capture only periods of unusually high vocal activity (Buckland et al. 2010). This kind of variation might be explained by a range of factors, including non‐uniform density, individual differences in vocal behavior, social dynamics (e.g., intra‐ and interspecific interactions and neighbor effects), or broader weather conditions (Hagens et al. 2018; Sobroza et al. 2021; Symes et al. 2022). To avoid these problems, we recommend that—when resources allow and the research question dictates (Wood et al. 2021)—long‐duration and large‐scale deployments be used to adequately account for this potential variability (Lawson et al. 2023; Pérez‐Granados and Schuchmann 2021; Piel 2018). If funding is a limiting factor, low‐cost recorders, or rotating recorders among sites, may be viable options (Hill et al. 2018; Sugai et al. 2020).

### 4.1 Limitations

Our study has some limitations. First, acoustic recordings were limited to a 4‐h sampling window following astronomical twilight. While the vocal activity observed within this period appears sufficient for most research purposes, vocal activity peaks may also occur outside this window. Second, data collection was limited to the wet season. Although day number within this period did not significantly predict vocal activity, indicating no intra‐seasonal trend, other sportive lemur studies indicate that vocal activity can be substantially lower during the dry season (Mandl et al. 2019). Long‐term studies are needed for a comprehensive assessment of seasonal patterns in *Lepilemur tymerlachsoni*. Third, environmental measurements were collected at a broad scale. Our weather station's location, approximately 3 km from the study area, means hourly temperature and rainfall data might not fully capture microclimate variations at each recording site, though we expect any spatial heterogeneity to be minimal given the small study area. Other unmeasured variables (e.g., humidity and cloud cover interactions with moonlight) could also influence activity (Linley et al. 2020; Pérez‐Granados and Schuchmann 2021). Ideally, contemporaneous environmental data, including additional variables, would be collected directly at recording sites, a practice that is becoming increasingly feasible with new low‐cost combined acoustic and environmental data loggers (Karlsson et al. 2021). Fourth, calls were treated as independent events in our analyses, but vocal activity sometimes occurred in non‐independent clustered bouts (e.g., from one or multiple individuals). Because we could not attribute calls to individuals—a common and perhaps unavoidable issue in PAM—we could not account for this dependence structure in the models (Gibb et al. 2019). This introduces a form of pseudoreplication, and a likely source of unexplained variability in our models, which can also increase the chances of Type 1 errors (Waller et al. 2013; Whitehouse et al. 2024). Future studies could explore advanced methods for acoustic identification of individual sportive lemurs, such as the unsupervised clustering techniques that show promise in Northern gray gibbons (*Hylobates funereus*) (Clink and Klinck 2020), to better account for clustered vocal activity in PAM recordings. Fifth, by using the number of detected and verified calls as a proxy for true vocal activity, our results are subject to potential detection biases; our results and recommendations would therefore benefit from validation across seasons and with improved detectors. Finally, while our results are consistent with predictions and generally agree with known behavior in other sportive lemurs, offering potential insights into congenerics and a framework for future acoustic surveys, caution should be exercised when generalizing these results because of species‐specific differences in the vocal repertoire, habitat, climate, ecology, and behavior (Rasoloharijaona et al. 2010).

## 5 Conclusion and Recommendations

Sportive lemurs are some of the most endangered primates in the world and urgent conservation action is needed, particularly to address data deficiencies in basic population metrics (Mittermeier et al. 2008, 2023). Our insights into the temporal and environmental drivers of vocal activity in *Lepilemur tymerlachsoni* establish a strong foundation for future PAM programs for this species, and provide a template for work on other members of its genus. This, we hope, will encourage the wider adoption of PAM and contribute to better conservation outcomes.

In summary, to improve survey design, detection probability, and population inferences, and to promote efficiency and cost‐effectiveness, PAM programs for *Lepilemur tymerlachsoni* should: (1) target ouah calls, preferably using automated call detection, and potentially also chuck calls with an improved detector, (2) focus recording on the fourth hour following astronomical twilight, (3) focus recording around full moon periods, (4) account for the effects of temperature (positive) and rainfall (negative) on vocal activity in the analysis, (5) ensure sufficient temporal and spatial replication in the deployment of audio recorders, and (6) consider conducting surveys during the wet season, which other studies suggest is the period of highest vocal activity for sportive lemurs.

## Conflicts of Interest

The authors declare no conflicts of interest.
